# Differential treatment responses to immune checkpoint inhibitor (ICI) therapy in a case of multiple primary malignancies: the programmed death ligand-1 (PD-L1) negative ureteral and lung metastasis from a clear cell renal cell carcinoma appearing after robotic-assisted partial nephrectomy progressed after ICI therapy, while synchronous PD-L1-positive primary lung squamous cell carcinoma responded very well to ICI therapy: a case report

**DOI:** 10.1186/s12957-023-02920-2

**Published:** 2023-02-06

**Authors:** Masayasu Urushibara, Kazuhiro Ishizaka, Noriyuki Matsutani, Mikiko Takahashi, Masakazu Nagata, Taisuke Okumura, Yuuki Matsumoto, Shinichiro Tatsuoka, Tsunehiro Nenohi, Takumasa Amemiya, Yohei Shimizu, Takeshi Shirakawa, Daisuke Kato

**Affiliations:** 1 Department of Urology, Teikyo University Hospital, Mizonokuchi, 5-1-1, Futago, Takatsu-ku, Kawasaki City, Kanagawa Prefecture 213-8507 Japan; 2 Department of Surgery, Teikyo University Hospital, Mizonokuchi, 5-1-1, Futago, Takatsu-ku, Kawasaki City, Kanagawa Prefecture 213-8507 Japan; 3 Department of Diagnostic Pathology, Teikyo University Hospital, Mizonokuchi, 5-1-1, Futago, Takatsu-ku, Kawasaki City, Kanagawa Prefecture 213-8507 Japan

**Keywords:** Multiple primary malignancies, Programmed death ligand-1, Immunohistochemistry, Immune checkpoint inhibitors, Renal cell carcinoma, Non-small cell lung carcinoma

## Abstract

**Background:**

Renal cell carcinoma (RCC) and non-small cell lung cancer (NSCLC) are representative malignancies that respond well to immune checkpoint inhibitors (ICIs). Research has been conducted to identify biomarkers, such as programmed death ligand-1 (PD-L1), that would allow the response to ICI therapy to be predicted; however, the complex tumor immune system consisting of both host and tumor factors may also exert an influence.

**Case presentation:**

Computed tomographic imaging (CT) incidentally revealed a left renal mass, and a left pulmonary nodule with multiple lymph node metastases (LNMs). Firstly, video-assisted thoracic surgery revealed a lung tumor invading the chest wall. Histologically, the findings of the tumor were consistent with squamous cell carcinoma (SCC), and immunohistochemistry (IHC) showed positive PD-L1 expression. The renal tumor was excised by robotic-assisted partial nephrectomy (RAPN). Histologically, the renal tumor showed the features of clear cell carcinoma (CCC). Four months after the RAPN, CT revealed left hydronephrosis caused by an enhancing ureteral tumor. Then, multiple right lung metastases appeared, and the left lung tumor increased. Following treatment including atezolizumab, the primary lung SCC and the multiple LNMs almost disappeared completely, while the ureteral and right lung metastases showed progression. The ureteral metastasis was resected by left open nephroureterectomy. Histology of the ureteral tumor revealed features consistent with CCC. Histological examination of the multiple right lung metastases that were resected by partial lobectomy via a small thoracic incision also revealed features consistent with CCC. Two months after nephroureterectomy, a solitary left lung metastasis was treated by nivolumab and ipilimumab. Six months after nephroureterectomy, the patient died of RCC. Further studies of specimens revealed that the tumor cells in the primary RCC and the ureteral and lung metastases showed negative results of IHC for PD-L1.

**Conclusions:**

The responses to ICI therapy of concomitant RCC and NSCLC were quite different. The PD-L1 expression status in individual tumors in cases of multiple primary malignancies (MPMs) may directly predict the response of each malignancy to ICI therapy, because the host immune system, which may affect the response to ICI therapy, could be the same in MPMs.

## Background

Only about 60 clinical cases of ureteral metastasis from renal cell carcinoma (RCC) have been reported in the literatures [1, 2]. Studies have shown that treatment with immune checkpoint inhibitors (ICIs) elicits more sustained responses and yields longer survivals in patients with a variety of malignancies, including advanced RCC [3] and non-small cell lung carcinoma (NSCLC) [4]. In a previously reported study, 271 (1%) out of 26,255 patients with carcinoma had multiple primary malignancies (MPMs), and moreover, 92 (34%) of these patients had synchronous malignancies [5]. With the increased life expectancy of humans and improvements in diagnostic methods, the number of patients with synchronous MPMs may be expected to increase. It might be possible to treat synchronous MPMs simultaneously shrunk with ICIs, which activate the host immune system against malignancy. Individual persons and tumors show different results of immunohistochemistry (IHC) for programmed death ligand-1 (PD-L1). Herein, we report a case in which the PD-L1-negative ureteral metastasis detected after robotic-assisted partial nephrectomy (RAPN) for early clinical stage RCC progressed rapidly after ICI therapy, while the almost synchronously detected PD-L1-positive primary advanced lung squamous cell carcinoma (SCC) with multiple lymph node metastases disappeared, with a sustained response, after ICI therapy.

## Case presentation

**Fig. 1:**
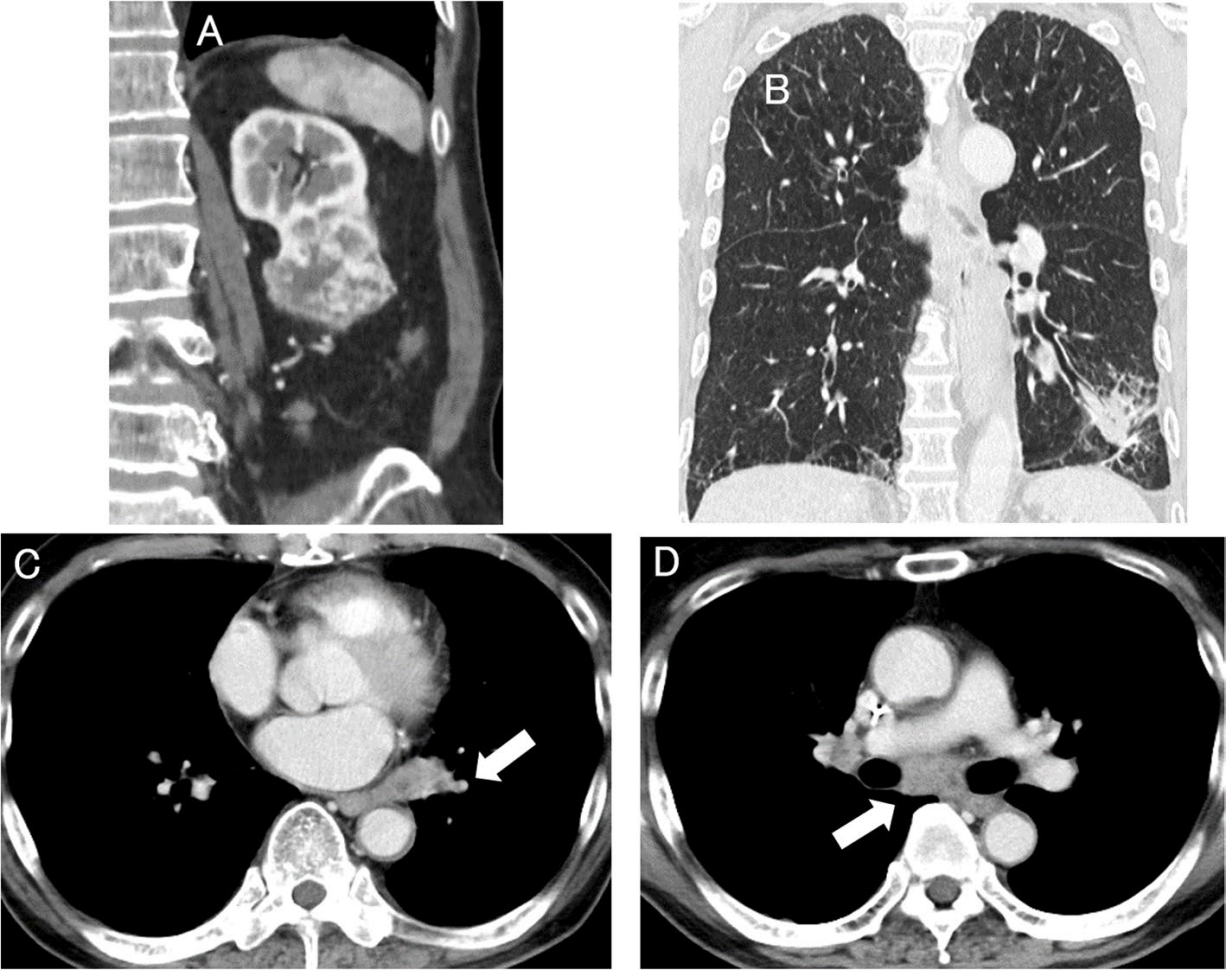
Contrast-enhanced computed tomographic image at the first visit. **A** The tumor in the lower pole of the left kidney, measuring about 50 mm in diameter, appears to protrude slightly into the peri-renal fat tissue. **B** The tumor was located in the lower portion of the left lung. The left hilar nodes (**C**) and nodes at the bronchial bifurcation (**D**) were enlarged (white arrow)

**Fig. 2:**
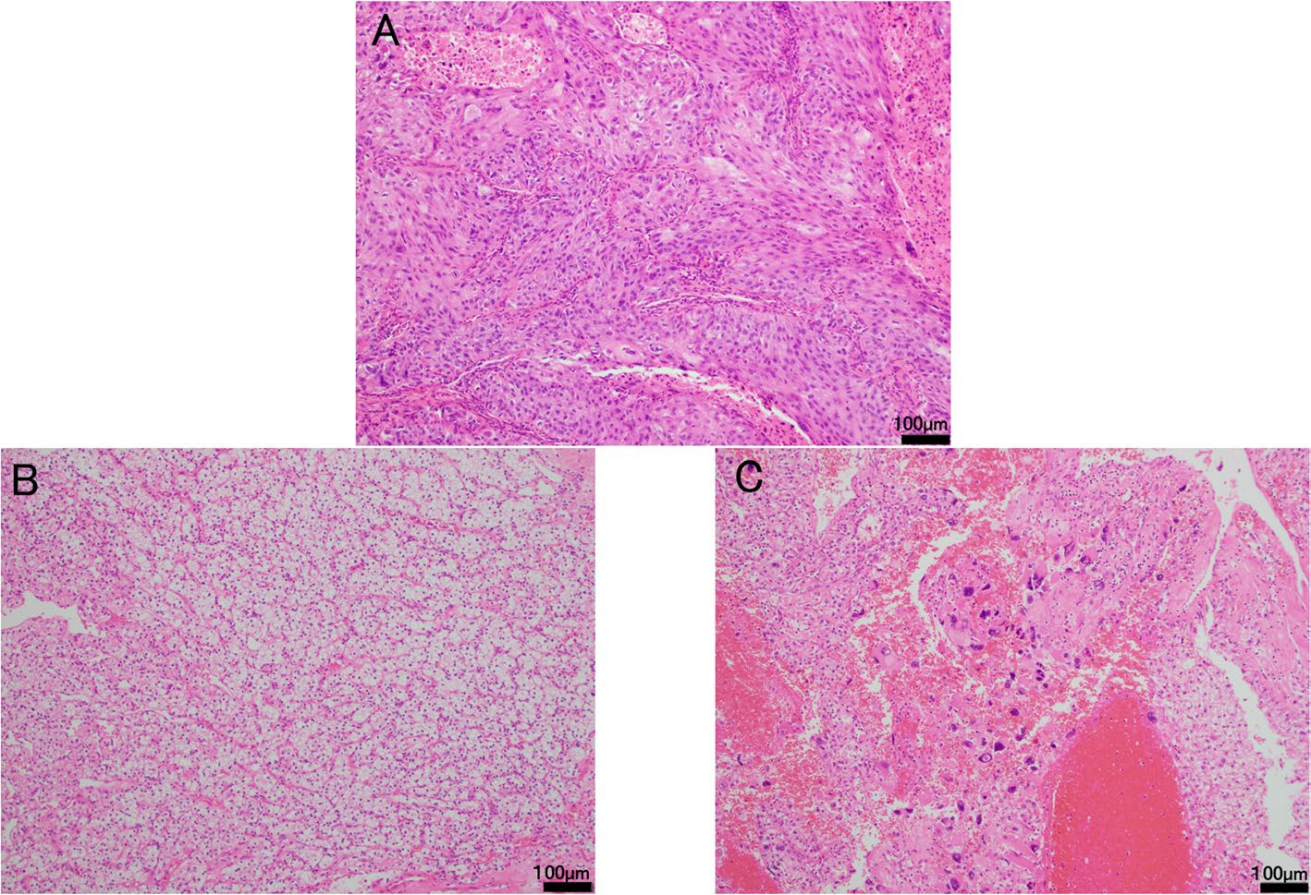
**A** The left lung squamous cell carcinoma (SCC) specimen obtained after resection by video-assisted thoracic surgery. Histological findings of the specimens obtained after robotic-assisted partial nephrectomy. **B** Clear cell carcinoma in the resected left primary renal tumor. Most cancer cells were classified into WHO/ISUP nuclear grade 2, but **C** there were also some partially grade 3 cancer cells. Hematoxylin–eosin stain (HE), reduced from 100 ×

**Fig. 3:**
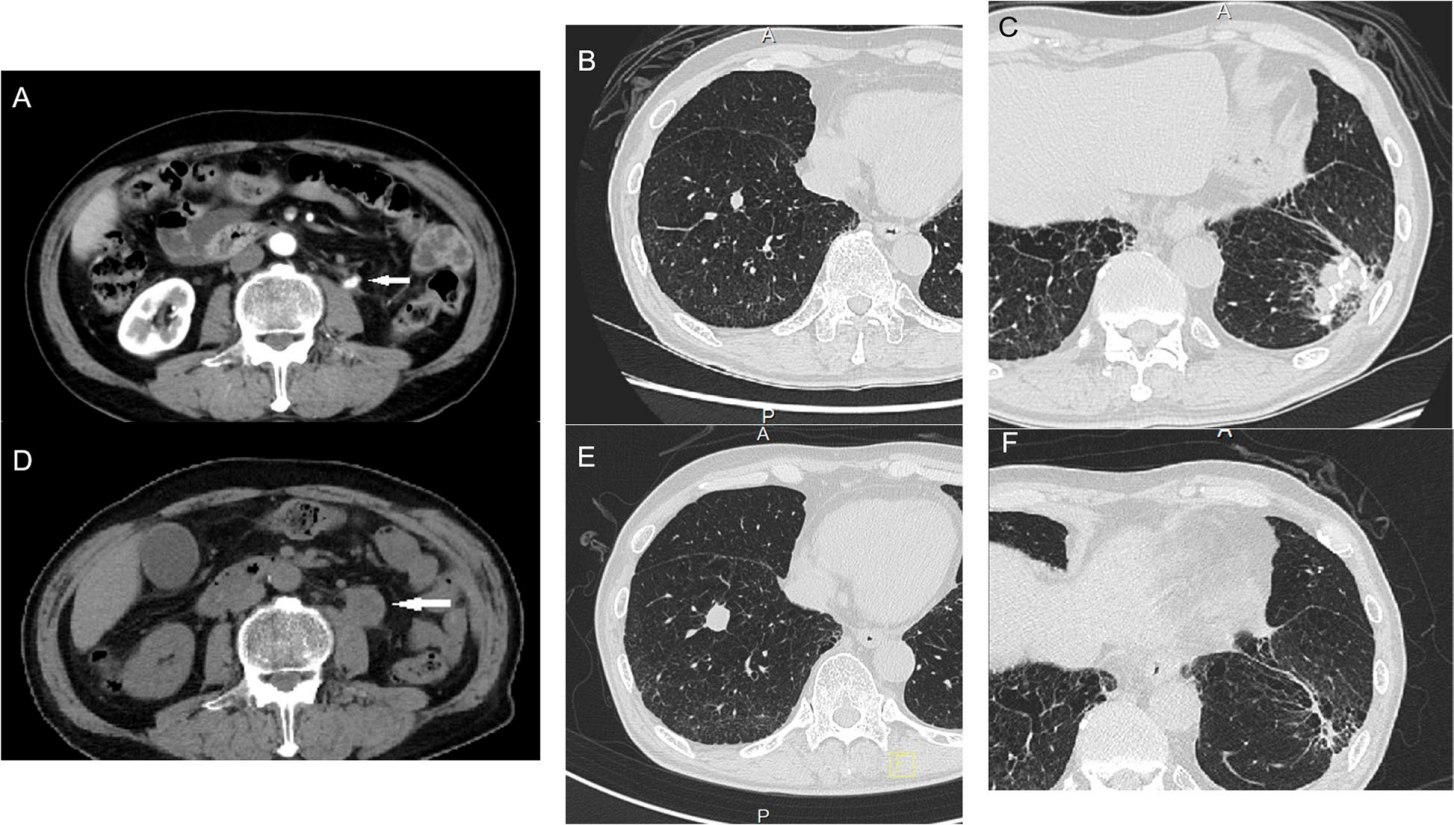
Contrast-enhanced computed tomographic images obtained before and non-enhanced computed tomographic images obtained after the combined multidrug therapy including atezolizumab. **A** Well-enhanced mass in the left upper ureter (white arrow), **B** right lung metastasis from the renal cell carcinoma (RCC), and **C** primary SCC in the left lung before the therapy. **D** The ureteral (white arrow) and **E** right lung metastasis from the RCC grew after the therapy, while **F** the primary SCC in the left lung almost disappeared

**Fig. 4:**
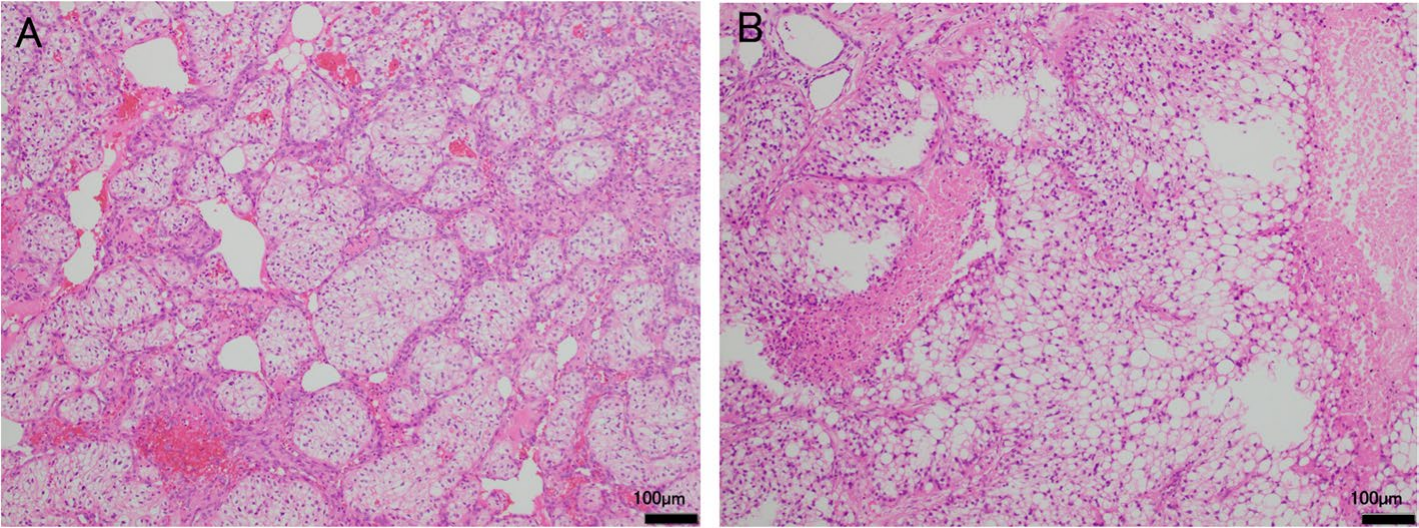
Histological findings after the combined multidrug therapy including atezolizumab. **A** Viable clear cell carcinoma (WHO/ISUP nuclear grade 2) in the left ureteral wall. HE, reduced from 100 × . **B** The right lung metastasis resected by partial lobectomy via a small thoracic incision also revealed vivid features of clear cell carcinoma. HE, reduced from 100x

**Fig. 5:**
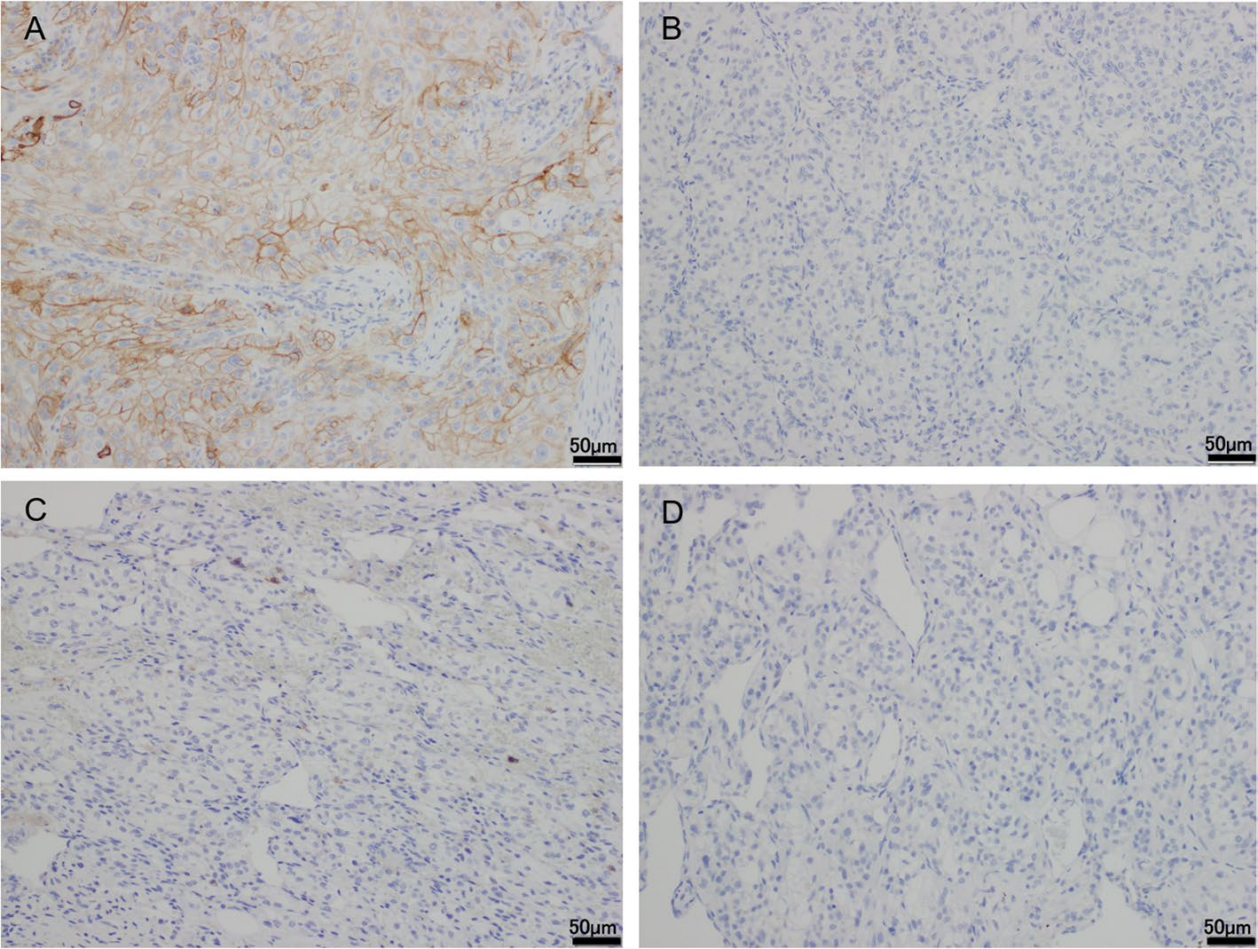
Programmed death-ligand 1 (PD-L1 clone 22C3) immunohistochemistry in **A** lung SCC, **B** the renal tumor, **C** ureteral metastasis, and **D** lung metastasis from the RCC. The tumor proportion score (TPS) in **B**, **C**, and **D** was < 1%, whereas that in **A** was 1–49%. PD-L1, reduced from 100 ×

A 78-year-old asymptomatic man with no significant past medical history, who had smoked 20 cigarettes a day for 50 years underwent preoperative computed tomographic imaging (CT) for chronic perforated otitis media; the imaging examination incidentally revealed an irregular tumor measured about 50 mm in diameter in the lower pole of the left kidney and also a large left lung tumor with multiple lymph node metastases, involving the nodes in the left pulmonary hilum, mediastinum, and bronchial bifurcation (Fig. 1A—D). The Karnofsky performance status was rated as 0. First of all, video-assisted thoracic surgery to obtain histological confirmation of lung metastasis from the RCC revealed invasion of the chest wall by the left lung tumor. On the other hand, histological examination of the left lung tumor showed features consistent with SCC, and IHC revealed a positive result for tumor PD-L1 expression (clone 22C3, tumor proportion score (TPS): 1–49%) (Fig. 2A, 5A). Positron emission tomography revealed supraclavicular lymph node metastases. The R.E.N.A.L. nephrometry score was 6 (maximal diameter: 2; exophytic properties: 1; nearness of the tumor to the collecting system: 1; location relative to the polar line was entirely below the lower polar line: 2) [6]. Three weeks after the lung surgery, the renal tumor was treated by intraperitoneal RAPN performed by an expert surgeon, and the postoperative course was uneventful. Histological examination of the left renal tumor revealed clear cell carcinoma (WHO/ISUP nuclear grade 3) invading the perirenal fat tissue, but neither invading the renal pelvis nor the surgical margin (Fig. 2B, C). No venous or lymphatic invasion was seen either. While additional treatment of the left lung carcinoma was necessary, the patient refused any treatment due to the intense burden posed by the repeated examinations and surgeries. Four months after the RAPN, the patient neither had hematuria nor positive urinary cytology, but CT revealed left hydronephrosis caused by a markedly enhancing upper ureteral tumor. Multiple right lung metastases appeared and the left lung tumor also increased in size (Fig. 3A–C). Considering the volumes of the tumors, treatment of the lung SCC seemed to take priority over that of the metastases from the RCC, which was categorized into the intermediate category according to the IMDC classification (the detection of neutrophilia and metastases within 1 year of the nephrectomy) [7]. Treatment with a combination of atezolizumab (1200 mg/body), nanoparticle albumin-bound paclitaxel (100 mg/m^2^), and carboplatin (area under the blood concentration–time curve 6) was administered by intravenous infusion of the drugs every 3 to 4 weeks for up to 4 cycles. The primary lung SCC with the lymph node metastases showed marked regression, while the patient developed persistent appetite loss. Moreover, 4 cycles of atezolizumab (1200 mg/body) alone were administered for maintenance by intravenous infusion every 4–6 weeks. By 6 months later, the left lung SCC with lymph node metastases had almost disappeared, whereas the left upper ureteral metastasis and right lung metastases from the RCC showed rapid progression (Fig. 3D–F). The patient showed a good performance status and the tumor volumes of all the metastatic tumors in the multiple organs seemed low, and therefore, open intraperitoneal nephroureterectomy was selected, even though treatment with other ICIs, tyrosine kinase inhibitors (TKIs), or a combination of the two was considered. Intraoperative exploration showed that the left upper ureteral tumor was tightly adherent to the mesenterium of the descending colon, and the left renal hilum was completely covered with hard bulky inflammatory tissues. Histological examination of the tumor in the ureteral wall compressing the lumen, which measured approximately 20 mm in diameter, revealed features consistent with clear cell carcinoma (WHO/ISUP grade 2) (Fig. 4A). Then, four right lung metastases measuring less than 20 mm in diameter were treated by partial lobectomy via a small thoracic incision. Histological examination of these tumors also revealed findings consistent with clear cell carcinoma (Fig. 4B). Two months after nephroureterectomy, a left lung metastasis in the lower lobe that appeared measuring about 10 mm in diameter seemed to be a metastasis from the RCC rather than a primary SCC, based on the previous clinical course. Considering the lack of efficacy of atezolizumab reported previously, it was thought that the lung metastasis would respond to TKI or TKI therapy combined with ICI therapy rather than to sequential ICI therapy [8]. However, after repeated attempts were made to obtain informed consent, the patient expressed a strong desire to continue to work rather than to live longer and rejected the therapies, including TKI therapy, considering the risk of adverse events, such as diarrhea and hand-foot syndrome that occur often and deteriorate the quality of life [9, 10]. Therefore, ICIs that exert their efficacy via a mechanism of action different from that of avelumab are desirable. The patient was treated with the combination of nivolumab (240 mg) and ipilimumab (1 mg/kg) administered concomitantly by intravenous infusion every 3 weeks [3]. A month later, the patient complained of back pain. Vertebral magnetic resonance imaging (MRI) performed 2 months later revealed a compression fracture without bone metastasis. After 2 cycles of this regimen, the patient needed hospitalization for general fatigue and appetite loss. The results of thyroid and adrenal hormone tests were almost within normal range, while the hypercalcemia associated with elevated levels of parathyroid hormone-related protein improved with subcutaneous injection of denosumab. While the lung SCC showed sustained response, CT revealed new metastatic lesions in the right lung and progression of the left lung metastasis, accompanied by liver metastasis, peritoneal carcinomatosis, and left retroperitoneal recurrence. Vertebral MRI showed multiple bone metastases. Radiotherapy was administered for the thoracic and lumbar vertebrae to relieve the severe back pain. One week after the hospitalization, the patient was diagnosed as having respiratory insufficiency caused by cancerous lymphangiomatosis with congestive heart failure. Six months after nephroureterectomy, the patient died of RCC. Subsequent studies revealed negative results of IHC (TPS < 1%) for PD-L1 in the primary RCC and ureteral and lung metastases from the RCC (Fig. 5B–D), these in RCC could have not routinely been performed in Japanese clinical diagnosis.

## Discussion and conclusions

Several studies have shown an association between the tumor PD-L1 expression status and the response rate to ICI therapy. In NSCLC patients, the tumor PD-L1 expression status appears to be associated with the overall survival (OS) following PD-L1 inhibitor treatment. Atezolizumab treatment was found to yield a significantly longer OS as compared with platinum-based chemotherapy in patients with NSCLC showing high PD-L1 expression, regardless of the histologic type [11]. The randomized phase III IMpower131 study suggested that addition of atezolizumab to carboplatin plus nab-paclitaxel provides progression-free survival (PFS) and OS benefit in patients with metastatic squamous NSCLC showing high tumor PD-L1 expression [12]. In patients with previously untreated advanced NSCLC showing PD-L1 expression in at least 50% of tumor cells, pembrolizumab therapy was associated with a significantly longer PFS and OS as compared with platinum-based combination chemotherapy (KEYNOTE-024 trial) [13].

While PD-L1 IHC and atezolizumab are available for the treatment of NSCLC, they have not been used for the treatment of RCC in Japan. While avelumab in combination with axitinib is available for the treatment of RCC, avelumab has not been used for the treatment of NSCLC in Japan. In two previous studies, the results of PD-L1 expression testing, including by IHC, in primary RCC were not found to be useful to predict the response to PD-L1 inhibitor therapy [14, 15]. The tumor PD-L1 expression status was not found to be associated with the responses to programmed death-1 (PD-1) inhibitor therapy in a phase III multicenter international randomized controlled trial, Checkmate 025, and nivolumab was approved for use as monotherapy for advanced clear cell RCC following failure of treatment with at least one TKI, with survival benefits observed irrespective of the tumor PD-L1 expression status [16]. In cases of clear cell RCC, response to anti PD-1 therapy is more common in patients with tumors showing PD-L1 expression; however, responses have also been observed among patients with tumors lacking in PD-L1 expression. Moreover, nivolumab treatment was not consistently associated with tumor PD-L1 expression in cases of metastatic RCC [17]. Surprisingly, the tumor PD-L1 expression status seemed to be associated with the responses to combined PD-L1 antibody plus TKI therapy. The PFS was significantly longer after treatment with the anti–PD-L1 inhibitor, avelumab, in combination with axitinib versus sunitinib as first-line therapy in patients with metastatic RCC with PD-L1-positive tumors in the JAVELIN Renal 101 trial (HR 0.61; *P* < 0.001) [18]. In the phase III IMmotion151 study (NCT02420821), atezolizumab plus bevacizumab therapy prolonged the PFS in patients across all risk groups with untreated metastatic RCC who showed positive tumor PD-L1 expression (HR 0.74; *P* = 0.0217) [19]. However, these results of the subgroup analysis revealed that the prognosis was good even in cases with negative tumor PD-L1 expression, although the underlying mechanism remains unclear [19, 20]. Possible explanations have been provided [21]. First, there were differences among the studies in the anti-PD-L1 monoclonal antibodies used for IHC, the staining technique, the definition of PD-L1-positive tumor, scoring increments, and number of biopsy sites in the patients. Second, because of intratumoral genetic heterogeneity, if the study sample is small, PD-L1 IHC cannot really reflect the tumor PD-L1 expression status. Third, undefined factors may contribute to the responses to ICI therapy. Therefore, the associations between the tumor PD-L1 expression status and the response to ICI therapy can become clearer if these biases are minimized or excluded.

It is likely that activation of the host immune system against malignancy by ICI therapy is useful in patients with MPMs, meta-analyses have shown the efficacy of ICI therapy in each of the malignancies. In synchronous MPMs accompanied by metastases, many factors (for example, tumor aggressiveness, tumor volume, stage, which malignancy is the metastasis from, the expected prognosis and damage by therapies of both tumors) would affect the treatment decision, such as surgery versus chemotherapy, and moreover, the surgical method and choice of drugs, for each malignancy. In our case, before the start of the combined multidrug treatment including atezolizumab, we thought that the survival prognosis would be associated to a greater degree with the response of the left lung carcinoma, because the tumor volume of the left lung advanced primary SCC with multiple lymph node metastases was larger than that of the ureteral metastasis and metastases in the right lung from the RCC that appeared after RAPN. Both tumors were considered as being likely to respond to atezolizumab. However, the ureteral and lung metastases from RCC grew during the ICI therapy, against our expectations. It is difficult to carry out a large-scale study of the efficacy of ICI therapy in cases of MPMs, because collecting a group of people with two or more malignancies within a definite period of time would be very difficult. Aoki reviewed 11 cases in the literature of ICI therapy for MPMs, including 3 of his own cases [22]. The first primary malignancies were solid tumor in 11 cases and leukemia in 3 cases. RCC was not a first malignancy in any of the cases, but was the second malignancy in one case of malignant melanoma [23]. The ICI drugs used were pembrolizumab in 7 cases, nivolumab in 5 cases, and ipilimumab in 1 case and atezolizumab (with bevacizumab) in 1 case. Same treatment responses in 8 cases were complete remission in 2 cases, partial response in 1 case, stable disease in 1 case, and progressive disease in 4 cases. Different responses to the first and second malignancies were observed in 6 cases (complete remission and partial response in 1 case, stable disease and partial response in 3 cases, stable disease and complete remission in 1 case, partial response and stable disease in 1 case). However, there were no cases of combined positive (complete remission or partial response) and negative (progressive disease) responses as in our case reported herein.

Then, how much does tumor PD-L1 IHC contribute to predicting the responses of each tumor to ICI therapy in patients with MPMs? Little is known about the PD-L1 expression statuses of tumors in patients with MPMs. To the best of our knowledge, only two reports have shown positive results of PD-L1 IHC in cases of MPMs. In the first, after cytotoxic chemotherapy of drugs, nivolumab was found to be ineffective against a lung adenocarcinoma (stage: cT1cN0M1a, stage IVA) that was immunohistochemically negative for PD-L1 expression (0%) [24]; on the other hand, a hypopharyngeal SCC (stage: cT2N1M0, stage III) that showed moderate PD-L1 positivity (30%) showed a remarkable response to nivolumab administration. In the second study, a lung SCC was found to show 90–100% positivity for PD-L1. The clinical stage was cT2bN3M1a, stage IVA, according to the TNM classification. Pembrolizumab as the initial therapy in this patient with MPMs resulted in a dramatic response of the lung SCC, even though administration was delayed for the treatment of its adverse events. Thereafter, sigmoid colon adenocarcinoma was treated by laparoscopic sigmoidectomy. The final pathological stage of the colon adenocarcinoma was pIIB (T4aN0M0) according to the TNM classification. The colon cancer cells were PD-L1-negative, with degenerative changes and necrosis [25]. In these studies, the tumor immune environment, which consists of various immune cells such as cytotoxic T cells, helper T cells, regulatory T cells, B cells, macrophages, dendritic cells, and natural killer cells that could be associated with the response to ICI therapy in cases of RCC [17, 26] and NSCLC [27], was not examined. Even for the same kind of malignancy, the tumor immune environments could differ among patients. In contrast, it was thought that the tumor immune environments are similar in each MPM patient, so that the tumor PD-L1 status may be more directly associated with the responses to ICI therapy in MPM patients than in non-MPM patients, without or minimizing the effect of tumor environmental immune.

Our findings in this case need to be interpreted with some caution. For example, the primary lung SCC may have responded to the other anti-cancer drugs than atezolizumab in our case. Because there is limited literature about the efficacy of ICI therapy in patients with MPMs, no definitive conclusions can be made, and further research is necessary. It is thought that the heterogeneity in the primary or metastatic tumor cells in cases of MPMs could cause variations in the results of PD-L1 IHC even within the same lesion. To the best of our knowledge, there are no reports of MPMs with PD-L1-negative tumors, so that it remains unknown if such tumors truly do not respond to ICI therapy. While we spotlighted on the expression status of PD-L1 in the tumors, other components of the host immune system might also play roles in the outcomes of patients with MPMs. Moreover, each tumor in cases of MPMs may have a local and/or systemic immune tumor environment that interacts with the tumor environment of other tumors. However, to the best of our knowledge, this is the first case report of MPMs with a ureteral metastasis from RCC and NSCLC that unexpectedly showed quite opposite responses to ICI therapy, which were later found to be strongly associated with the respective tumor PD-L1 statuses.

In conclusion, we have reported a relatively rare case of ureteral metastasis from a PD-L1-negative RCC with primary PD-L1-positive SCC of the lung. Before the ICI treatment, considering the tumor volumes, it seemed that the prognosis of our patient would depend more on the treatment response of the primary advanced lung SCC with lymph node metastases than on that of the ureteral and lung metastases from the RCC. However, after the ICI therapy (PD-L1 inhibitor followed by PD-1 and cytotoxic T-lymphocyte antigen 4 inhibitors), unexpectedly, the PD-L1-negative metastatic RCC progressed rapidly, while the PD-L1-positive SCC of the lung showed sustained good response. Our result may suggest that the tumor PD-L1 status in MPMs is directly predictive of the responses to ICI therapy.

## Data Availability

All data generated or analyzed during this study are included in this article.

## Abbreviations

CT: Computed tomographic imaging
ICI: Immune checkpoint inhibitor
IHC: Immunohistochemistry
IMDC: International Metastatic RCC Database Consortium
ISUP: International Society of Urological Pathology
MPMs: Multiple primary malignancies
MRI: Magnetic resonance imaging
NSCLC: Non-small cell lung carcinoma
OS: Overall survival
PD-1: Programmed death-1
PD-L1: Programmed death ligand-1
PFS: Progression free survival
RAPN: Robotic-assisted partial nephrectomy
RCC: Renal cell carcinoma
SCC: Squamous cell carcinoma
TKI: Tyrosine kinase inhibitor
TPS: Tumor proportion score
WHO: World Health Organization
